# Genome-wide association study reveals new loci for yield-related traits in Sichuan wheat germplasm under stripe rust stress

**DOI:** 10.1186/s12864-019-6005-6

**Published:** 2019-08-08

**Authors:** Xueling Ye, Jian Li, Yukun Cheng, Fangjie Yao, Li Long, Yuqi Wang, Yu Wu, Jing Li, Jirui Wang, Qiantao Jiang, Houyang Kang, Wei Li, Pengfei Qi, Xiujin Lan, Jian Ma, Yaxi Liu, Yunfeng Jiang, Yuming Wei, Xianming Chen, Chunji Liu, Youliang Zheng, Guoyue Chen

**Affiliations:** 1 Triticeae Research Institute, Sichuan Agricultural University, Wenjiang, Chengdu, Sichuan 611130 People’s Republic of China; 2 College of Agronomy, Sichuan Agricultural University, Wenjiang, Chengdu, Sichuan 611130 People’s Republic of China; 3 US Department of Agriculture, Agricultural Research Service, Wheat Health, Genetics and Quality Research Unit; and Department of Plant Pathology, Washington State University, Pullman, WA 99164-6430 USA; 4 CSIRO Agriculture and Food, St Lucia, Queensland 4067 Australia

**Keywords:** Wheat, 55 K SNP, Genome-wide association study, Yield-related traits, Stripe rust

## Abstract

**Background:**

As one of the most important food crops in the world, increasing wheat (*Triticum aestivum* L.) yield is an urgent task for global food security under the continuous threat of stripe rust (caused by *Puccinia striiformis* f. sp. *tritici*) in many regions of the world. Molecular marker-assisted breeding is one of the most efficient ways to increase yield. Here, we identified loci associated to multi-environmental yield-related traits under stripe rust stress in 244 wheat accessions from Sichuan Province through genome-wide association study (GWAS) using 44,059 polymorphic markers from the 55 K single nucleotide polymorphism (SNP) chip.

**Results:**

A total of 13 stable quantitative trait loci (QTLs) were found to be highly associating to yield-related traits, including 6 for spike length (SL), 3 for thousand-kernel weight (TKW), 2 for kernel weight per spike (KWPS), and 2 for both TKW and KWPS, in at least two test environments under stripe rust stress conditions. Of them, ten QTLs were overlapped or very close to the reported QTLs, three QTLs, *QSL.sicau-1AL*, *QTKW.sicau-4AL*, and *QKWPS.sicau-4AL.1*, were potentially novel through the physical location comparison with previous QTLs. Further, 21 candidate genes within three potentially novel QTLs were identified, they were mainly involved in the regulation of phytohormone, cell division and proliferation, meristem development, plant or organ development, and carbohydrate transport.

**Conclusions:**

QTLs and candidate genes detected in our study for yield-related traits under stripe rust stress will facilitate elucidating genetic basis of yield-related trait and could be used in marker-assisted selection in wheat yield breeding.

**Electronic supplementary material:**

The online version of this article (10.1186/s12864-019-6005-6) contains supplementary material, which is available to authorized users.

## Background

Wheat (*Triticum aestivum* L.) is one of the most important food crops in the world and provides 20% of calories consumed by humans [1]. Producing enough wheat for the growing population is one of the vital tasks for food security. However, abiotic and biotic stresses are among the greatest challenges to wheat production. As one of the most destructive wheat diseases in the world, stripe rust that is caused by fungus *Puccinia striiformis* Westend. f. sp. *tritici* Erikss. (*Pst*) is a serious threat to wheat production [2]. Therefore, improving wheat yield under stripe rust stress is extremely urgent. Thus, identifying loci associated with yield-related traits under stripe rust stress may provide favourable alleles and their useful markers for breeding wheat cultivars with high yield in combination with stripe rust resistance.

The productive spike number per unit area, kernel number per spike (KPS) and thousand-kernel weight (TKW) are key components of wheat yield. The productive spike number per unit area mainly depends on the fertile tiller number (FTN). Most spike-related traits, such as spike length (SL), spikelet number per spike (SlPS), kernel number per spikelet (KPSl) and the spikelet compactness (SlC), affect the KPS and thus also affect the yield [3–5]. Many studies have showed that the SL has a positive correlation with KPS and SlPS [3, 4, 6, 7]. Moreover, Mohsin et al. [8] reported that the SL and the KPS had a positive effect on grain yield. Würschum et al. [7] demonstrated that KPSl was positively correlated with the KPS. The SlC is positively correlated with SlPS, but negatively related to SL [3, 6, 9]. The TKW, which depends on the kernel weight, is associated with the accumulation of starch produced by photosynthesis [10, 11]. Therefore, wheat yield is a complex quantitative trait contributed by many morphological, physiological and biochemical components, all of which can be improved to increase the yield directly or indirectly.

Genome-wide association study (GWAS) is a powerful tool to identify loci associated to target traits based on linkage disequilibrium (LD) using natural populations. It is a rapid and cost-effectiveness way to detect target markers for marker-assisted breeding. GWAS was first used in human research and has made great contributions to identify genes associated to human diseases [12–16]. The GWAS approach has been widely used in plant and animal research [17–21]. For wheat, GWAS has been successfully used for identifying quantitative trait loci (QTLs) for disease resistance and yield [22–25]. The release of the high-quality genome reference IWGSC RefSeq v1.0 [26] has provided great assistance to detect linked markers and candidate genes for target traits. The availability of marker arrays for high throughput genotyping is a key for GWAS. There are many SNP arrays have been developed for wheat, such as 9 K, 35 K, 55 K, 90 K, 660 K and 820 K. These arrays are able to provide high-density maps for detecting loci associated with target traits.

In the present study, 244 wheat accessions (including 79 landraces and 165 cultivars) from Sichuan Province, China were genotyped by using the wheat 55 K SNP Array [27]. Based on multi-environmental yield-related traits data under stripe rust stress, a GWAS was conducted to identify the associated loci for yield-related traits, such as FTN, SL, SlPS, Kernel weight per spike (KWPS), TKW and SlC. The research further analysed the genetic architecture of the yield-related traits, provide molecular markers to use in genome selection for wheat high-yield breeding and may provide new insights for genetic dissection of other complex quantitative traits in wheat.

## Results

### Phenotypic characterization of eight yield-related traits

**Fig. 1:**
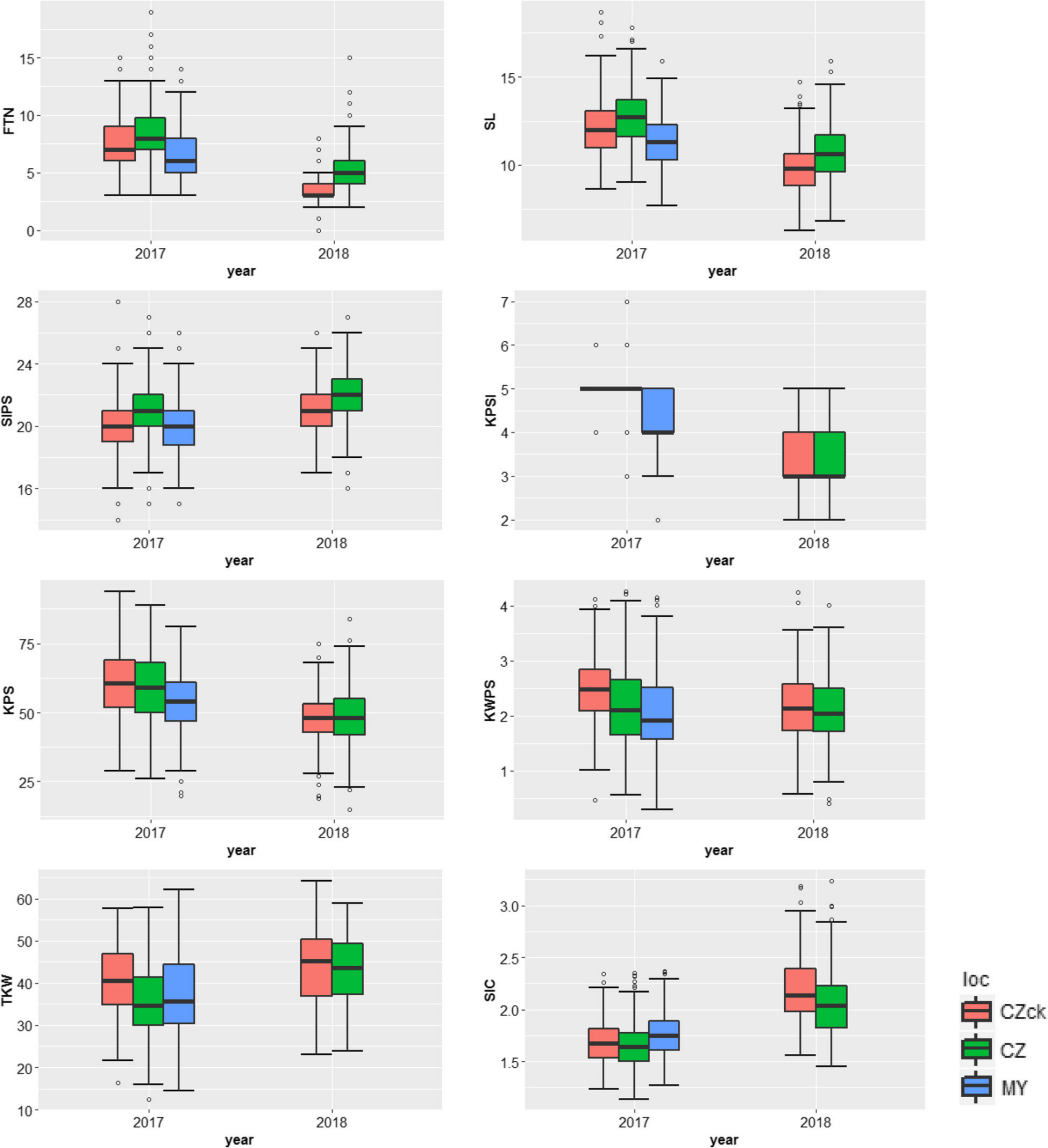
The box plots of eight yield-related traits in multiple environments. It clearly showed that the genotype-by-year interaction variance was significant for all yield-related traits we measured except for KWPS. FTN, Fertile tiller number; SL, Spike length; SlPS, Spikelet number per spike; KPSl, Kernel number per spikelet; KPS, Kernel number per spike; KWPS, Kernel weight per spike; TKW, Thousand-kernel weight; SlC, Spikelet compactness. CZck = Chongzhou without *Puccinia striiformis* f. sp. *tritici* (*Pst*) inoculation; CZ = Chongzhou with *Pst* inoculation; MY = Mianyang with *Pst* inoculation

The yield-related traits were collected from Chongzhou in 2017, 2018 (CZ17, CZ18), Mianyang in 2017 (MY17) under stripe rust stress, and Chongzhou in 2017, 2018 without *Pst* inoculation as control (CZ17ck, CZ18ck). The materials in CZ17ck and CZ18ck were set as control without inoculating with *Pst*. The materials in CZ17, MY17 and CZ18 were inoculated with mixed *Pst* isolates. The Pearson correlation coefficient analysis showed the significant correlations among the five environments when the seven yield-related traits except FTN were analysed separately. FTN had significant correlation among the four test environments except for CZ18 (Additional file 1). Shown as Fig. 1, the genotype-by-year interaction was significant for all measured yield-related traits except KWPS, while the genotype-by-location interaction was not significant. The FTN, SL, KPSl and KPS in 2017 all performed better than in 2018, while the SlPS, TKW and SlC all performed better in 2018 than in 2017. Moreover, the plants in CZ17 and MY17 showed lower KPS, KWPS and TKW than in CZ17ck, and the plants in CZ18 showed lower KWPS, TKW and SlC than in CZ18ck.

The phenotypic variations of eight yield-related traits under stripe rust stress were determined based on their best linear unbiased prediction (BLUP) values (Table 1). FTN and KPSl ranged from 6 to 9 and from 3.7 to 4.3, respectively. SL ranged from 9.3 to 14.1 cm. The ranges of SlPS and KPS were 18 to 25 and 41 to 62, respectively. The lowest KWPS was 1.21 g (g) and TKW 24.26 g, while the highest KWPS was 2.99 g and TKW 52.02 g. The maximal SlC was 2.53, and the minimum value was 1.54. In addition, SlPS, TKW and SlC had high heritabilities (0.78, 0.80 and 0.86, respectively) whereas FTN and KPSl had relatively low heritabilities (0.37 and 0.31, respectively). The Shannon-Weaver diversity index analysis showed that KPS (*H′* = 0.86) and TKW (*H′* = 0.85) exhibited relatively high diversity compared to FTN (*H′* = 0.68) and SlPS (*H′* = 0.67).

**Table 1:** The phenotypic variations for 244 wheat accessions under stripe rust stress based on BLUP values

Trials	FTN	SL (cm)	SlPS	KPSl	KPS	KWPS (g)	TKW (g)	SlC
Min	6	9.3	18	3.7	41	1.21	24.26	1.54
Max	9	14.1	25	4.3	62	2.99	52.02	2.53
Mean	6.8	11.6	21	4.0	53	2.09	39.54	1.88
STDEV	0.64	0.85	1.16	0.11	3.94	0.30	5.68	0.17
CV	0.09	0.07	0.06	0.03	0.07	0.14	0.14	0.09
*H* ^*2*^	0.37	0.69	0.78	0.31	0.51	0.59	0.80	0.86
*H′*	0.68	0.79	0.67	0.79	0.86	0.80	0.85	0.78

*BLUP* The best linear unbiased prediction

*FTN* Fertile tiller number, *SL* Spike length, *SlPS* Spikelet number per spike, *KPSl* Kernel number per spikelet, *KPS* Kernel number per spike, *KWPS* Kernel weight per spike, *TKW* Thousand-kernel weight, *SlC* Spikelet compactness, *cm* centimetre, *g* gram

*STDEV* Standard deviation, *CV* Coefficient of variation, *H*^*2*^, The broad sense heritability; *H′*, The Shannon-Weaver diversity index

### Phenotypic differences between landraces and cultivars under stripe rust stress

The 244 entries consisted of 79 landraces and 165 cultivars. The *t*-test identified significant differences between landraces and cultivars in FTN, SlPS, KPSl, KWPS, TKW and SlC under stripe rust stress based on the BLUP values (Table 2). The mean BLUP value of the landrace group was significantly higher than that of the cultivar group for FTN (7.3), SlPS (21) and SlC (1.95), whereas the cultivar group exhibited significant higher KPSl (4.1), KWPS (2.19) and TKW (42.14) values than the landrace group (Table 2). In addition, analysis of the Shannon-Weaver diversity indices showed that the landrace group exhibited higher phenotypic diversity in FTN, SL, SlPS and SlC than the cultivar group, whereas the latter group had higher diversity in KPSl, KWPS and TKW (Table 2).

**Table 2:** The phenotypic variations between landraces and cultivars under stripe rust stress based on BLUP values

Trials	FTN*		SL (cm)		SlPS*		KPSl*		KPS		KWPS (g) *		TKW (g) **		SlC*	
	Landrace	Cultivar	Landrace	Cultivar	Landrace	Cultivar	Landrace	Cultivar	Landrace	Cultivar	Landrace	Cultivar	Landrace	Cultivar	Landrace	Cultivar
Min	6	6	9.3	9.7	19	18	3.7	3.7	41	43	1.21	1.44	24.26	31.65	1.54	1.58
Max	9	7	14.1	13.9	25	23	4.2	4.3	62	61	2.33	2.99	40.44	52.02	2.53	2.33
Mean	7.3	6.5	11.3	11.7	21	20	4.0	4.1	54	53	1.89	2.19	34.12	42.14	1.95	1.84
STDEV	0.56	0.50	0.93	0.79	1.19	1.06	0.11	0.10	4.09	3.84	0.20	0.30	2.87	4.79	0.20	0.14
CV	0.08	0.08	0.08	0.07	0.06	0.05	0.03	0.03	0.08	0.07	0.10	0.14	0.08	0.11	0.10	0.08
*H′*	0.59	0.50	0.81	0.76	0.69	0.61	0.70	0.79	0.85	0.85	0.50	0.82	0.44	0.75	0.87	0.70

*BLUP* The best linear unbiased prediction

*FTN* Fertile tiller number, *SL* Spike length, *SlPS* Spikelet number per spike, *KPSl* Kernel number per spikelet, *KPS* Kernel number per spike, *KWPS* Kernel weight per spike, *TKW* Thousand-kernel weight, *SlC* Spikelet compactness, *cm* centimetre, *g* gram

*STDEV* Standard deviation, *CV* Coefficient of variation; *H′*, The Shannon-Weaver diversity index

*, Significant at *p* < 0.05; **, Significant at *p* < 0.01

### Correlations among yield-related traits under stripe rust stress and stripe rust reaction

**Fig. 2:**
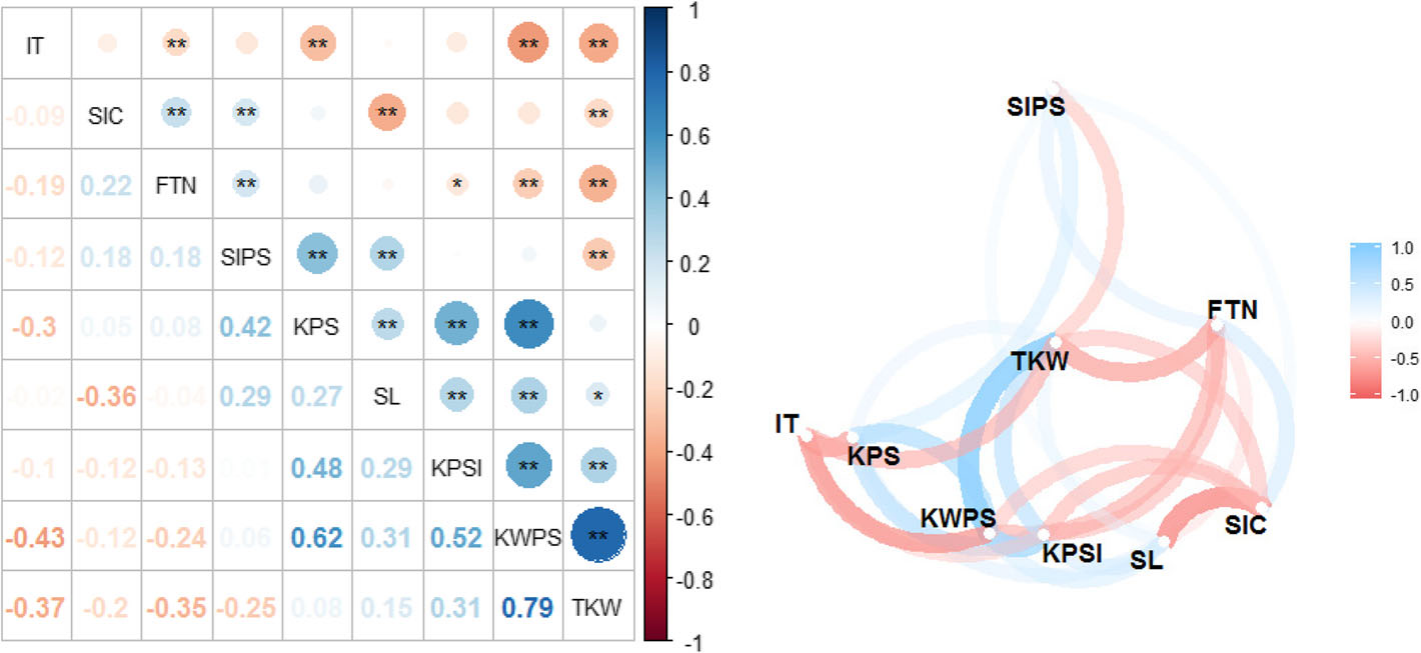
The correlations matrix and network analysis among eight yield-related traits and infection type (IT). FTN, Fertile tiller number; SL, Spike length; SlPS, Spikelet number per spike; KPSl, Kernel number per spikelet; KPS, Kernel number per spike; KWPS, Kernel weight per spike; TKW, Thousand-kernel weight; SlC, Spikelet compactness. *, Significant at *p* < 0.05; **, Significant at *p* < 0.01

Pearson correlation coefficient analysis among the yield components and stripe rust reaction measured as IT (Additional file 2) showed that IT was significantly negatively correlated with FTN, KPS, KWPS and TKW; SlC was significantly negatively correlated with SL and TKW; FTN negatively correlated with KPSl, KWPS and TKW; and SlPS negative correlated with TKW (Fig. 2). Positive correlation was detected between SlC with FTN and SlPS; between FTN and SlPS; between SlPS with KPS and SL; and among KPS, SL, KPSl, KWPS and TKW except between TKW and KPS (Fig. 2). In addition, the correlation network analysis showed a very strong correlation between KWPS and TKW (0.79), and between KPS and KWPS (0.62) (Fig. 2).

### The impact of stripe rust on yield-related traits

The Pearson correlation coefficient analysis showed IT was negatively correlated with FTN, KPS, KWPS and TKW. In order to further understand the stripe rust effects on yield traits, we compared the control plots (CZ17ck and CZ18ck) and the inoculated plots (MY17, CZ17, and CZ18) (Table 3). The control plots had higher KPS, KWPS and TKW than inoculated plots, whereas the inoculated plots had higher FTN than the control plots (Table 3). Based on the IT data in the inoculated fields, of the 244 accessions, 169 were resistant and 75 susceptible (Additional file 2). The resistant accessions exhibited higher FTN, KPS, KWPS, and TKW than the susceptible accessions in both inoculated and non-inoculated plots (Table 3). However, compared with the non-inoculated plots, FTN increased by 15.5 and 15.4% for the resistant and susceptible accessions inoculated with *Pst*, respectively. In addition, with *Pst* inoculation, KPS, KWPS and TKW of resistance accessions were reduced by 1.5, 8.5 and 5.6%, and KPS, KWPS and TKW of susceptible ones reduced by 2.7, 11.8 and 10.2%, respectively (Table 3).

**Table 3:** The difference in yield-related traits between resistant and susceptible accessions with or without *Pst* inoculation

Traits	Type	Range		Mean values		Difference^c^
		Control^a^	Inoculation^b^	Control^a^	Inoculation^b^	
FTN**	R	3–11	6–9	5.94	6.86	+ 15.5%
	S	4–10	6–8	5.65	6.52	+ 15.4%
SL (cm)	R	7.7–15.7	9.3–13.9	10.96	11.57	–
	S	8.0–16.0	9.8–14.1	10.78	11.51	–
SlPS	R	17–25	18–23	20.85	20.80	–
	S	18–24	18–25	20.49	20.39	–
KPSl	R	3.0–5.5	3.7–4.3	4.18	4.05	–
	S	3.0–5.0	3.7–4.3	4.10	4.03	–
KPS**	R	28–75	43–62	54.53	53.72	−1.5%
	S	30–72	41–60	52.49	51.09	−2.7%
KWPS (g)**	R	0.84–3.61	1.32–2.99	2.36	2.16	−8.5%
	S	0.68–3.36	1.21–2.52	2.20	1.94	−11.8%
TKW (g)**	R	24.91–61.23	28.27–52.02	42.95	40.54	−5.6%
	S	23.38–54.88	24.26–45.26	41.54	37.31	−10.2%
SlC	R	1.44–2.76	1.54–2.53	1.91	1.89	–
	S	1.50–2.44	1.58–2.33	1.90	1.86	–

*FTN* Fertile tiller number, *SL* Spike length, *SlPS* Spikelet number per spike, *KPSl* Kernel number per spikelet, *KPS* Kernel number per spike, *KWPS* Kernel weight per spike, *TKW* Thousand-kernel weight, *SlC* Spikelet compactness, *cm* centimetre, *g* gram

*R* Resistant materials, *S* Susceptible materials

^a^,the locations without *Pst* inoculation;^b^, the locations with *Pst* inoculation.^c^, comparing with control, the increase (+) or decrease (−) percentage of yield-related traits under stripe rust stress

**,the significant difference in the traits between control and inoculation at *p* < 0.01

### Genome-wide association analyses

**Fig. 3:**
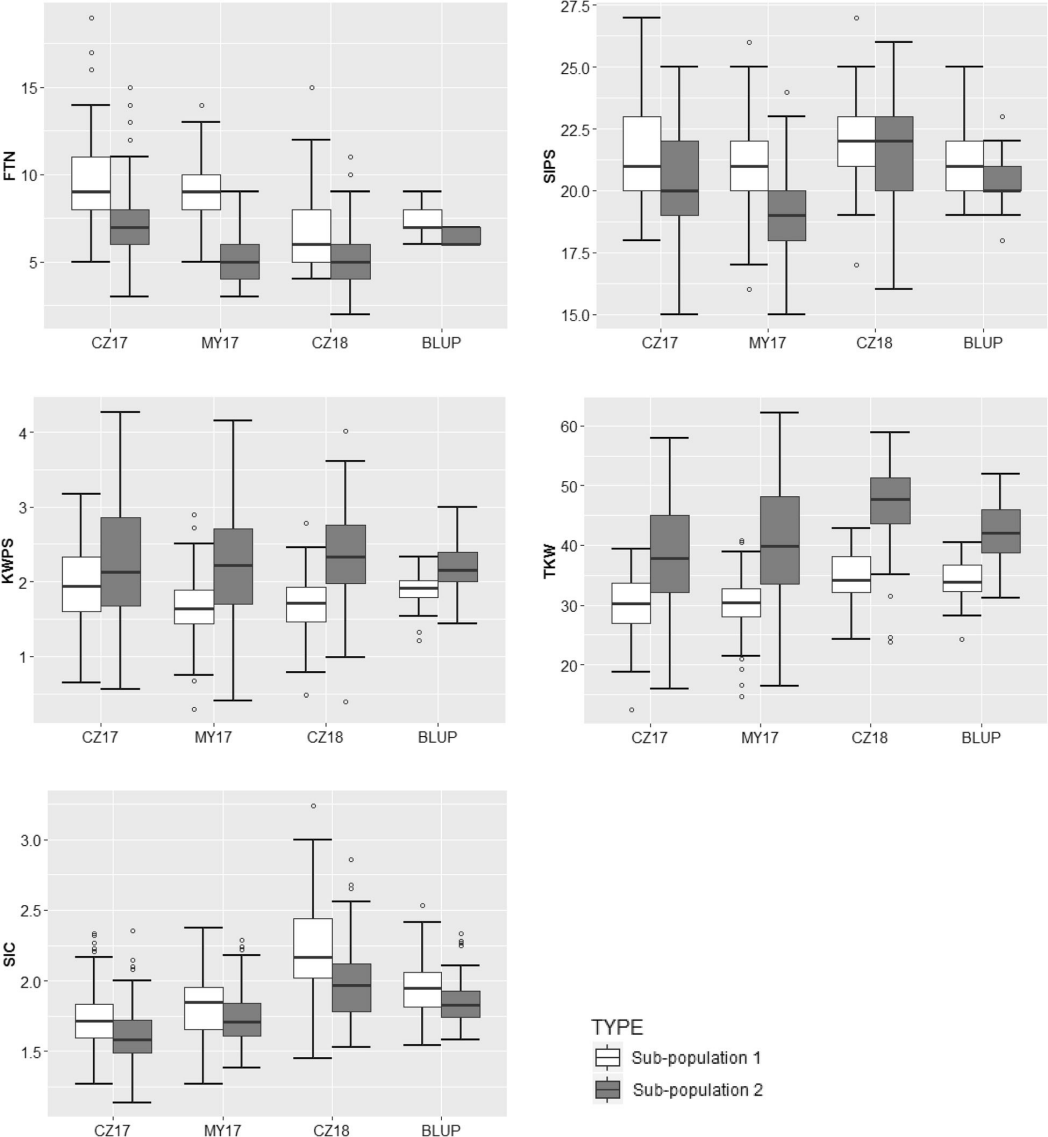
Significant difference in five yield-related traits under stripe rust stress between two sub-populations based on Q-matrix. FTN, Fertile tiller number; SlPS, Spikelet number per spike; KWPS, Kernel weight per spike; TKW, Thousand-kernel weight; SlC, Spikelet compactness. CZ17 = Chongzhou 2017; MY17 = Mianyang 2017; CZ18 = Chongzhou 2018; BLUP, the best linear unbiased prediction

Based on the genotyping data generated using the 55 K SNP array (Affymetrix Axiom Wheat55K), a total of 44,059 high-quality SNP markers were selected for genetic variation (Additional file 3) [27]. The previous result of the analysis of population structure (Q-matrix) showed the optimal ΔK value was 2, indicating that the 244 accessions could be divided into 2 sub-populations. Sub-population 1 harboured 78 accessions, including 77 landraces and one cultivar. Sub-population 2 contained 166 accessions, including 164 cultivars and 2 landraces [27]. Significant differences were observed in the BLUP values of five yield-related traits (FTN, SlPS, KWPS, TKW and SlC) between two sub-populations. The sub-population 1 mainly contained landraces exhibited higher FTN, SlPS and SlC than those in sub-population 2, while the accessions in sub-population 2 showed higher KWPS and TKW than those in sub-population 1 (Fig. 3, Additional file 4). The LD half decay distance was 2.12 Mb based on the *r*^*2*^ values between significant pairs of intra-chromosomal SNP markers with physical distances [27]. Significant associated loci within a genomic region of 2.12 Mb or less on the same chromosome were treated as a same QTL.

**Fig. 4:**
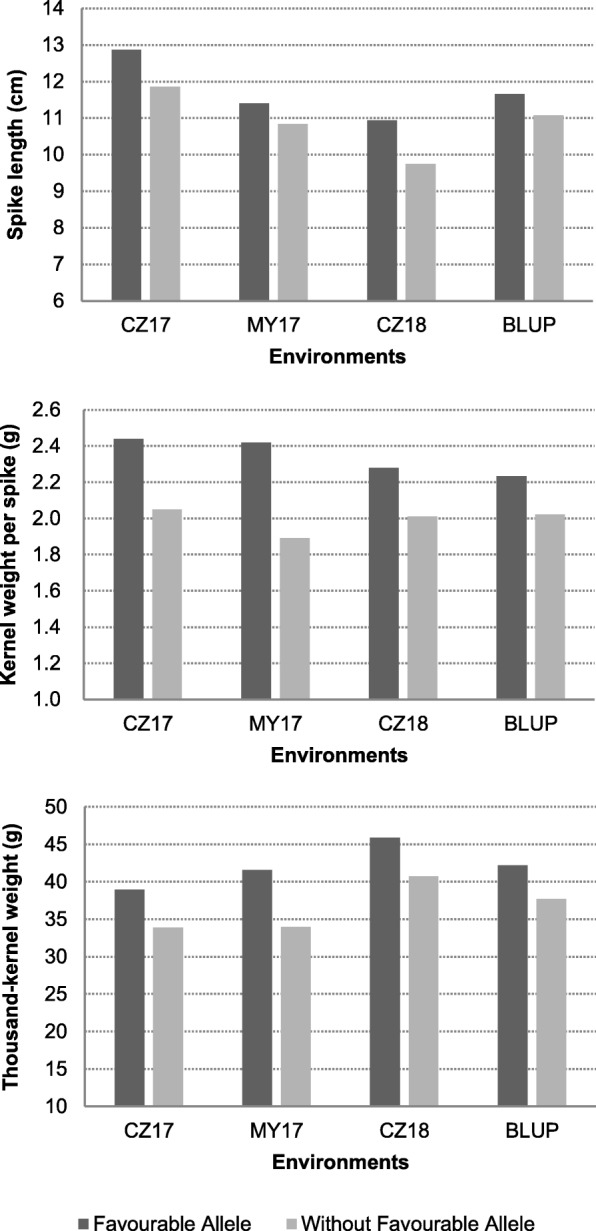
The difference in traits between accessions with and without favourable allele were displayed as histogram. The accessions with favourable allele showed higher mean values of spike length, kernel weight per spike and thousand-kernel weight than that without favourable allele in three environments under stripe rust stress and BLU*P* values. CZ17 = Chongzhou 2017; MY17 = Mianyang 2017; CZ18 = Chongzhou 2018; BLUP, the best linear unbiased prediction

**Fig. 5:**
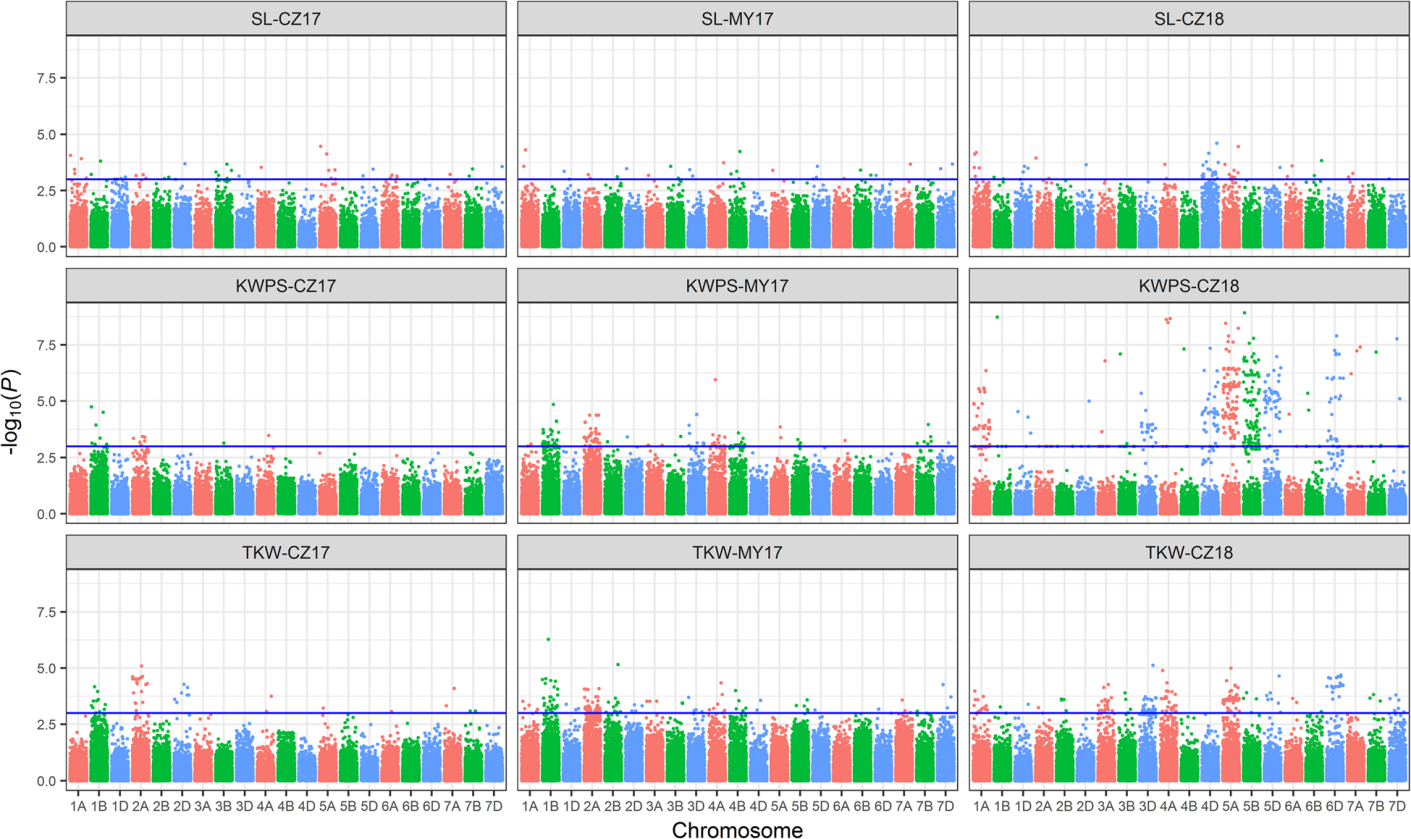
The *P* values of associated loci with yield-related traits under stripe rust stress exhibited as Manhattan plots. The associated loci with SL, KWPS and TKW in three test environments were displayed as Manhattan plots with *P* values across 21 wheat chromosomes. The significant associated loci were considered as –log_10_(*P*) > 3 which upper the blue lines. SL, Spike length; KWPS, Kernel weight per spike; TKW, Thousand-kernel weight. CZ17 = Chongzhou 2017; MY17 = Mianyang 2017; CZ18 = Chongzhou 2018

Subsequently, GWAS was conducted to identify loci associated with yield-related traits in three environments under stripe rust stress based on the MLM model with Q + K as covariates. We identified 7, 9 and 6 high confidence markers (−log_10_ *P* > 3) associated with SL, TKW and KWPS, respectively. Of them, four markers associated with both TKW and KWPS (Table 4). All of these high confidence markers were detected in at least two of the environments. Moreover, 16 favourable SNP alleles were detected (Table 4, Additional file 5). Accessions possessing the favourable alleles performed better in SL, KWPS and TKW than those did not have the favourable alleles (Fig. 4). Shown as Fig. 5, the loci with high confidences of association in the three environments were displayed as Manhattan plots with *P* values across the 21 wheat chromosomes. Based on the LD distance of 2.12 Mb, the associated loci were determined as 13 QTLs (Table 4). The six QTLs associated with SL were mapped on chromosome 1AL, 2AL, 2DS, 4AS and 5AL, three QTLs associated with TKW were located on chromosome 1BL, 2AS, and 4AL, two QTLs associated with KWPS were all mapped on chromosome 4AL, two QTLs associated with both KWPS and TKW were mapped on chromosome 1BL and 2AS. The two QTLs associated with KWPS located on chromosome 4AL explained up to 20% phenotypic variation, and the QTL on 1AL associated with SL also explained high phenotypic variation, ranging from 8.3 to 10.6% and was detected in all three environments. Moreover, comparing with the physical locations of reported QTLs or genes associated with SL, KWPS and TKW based on the reference RefSeq v1.0 [26], three of the QTLs were potentially novel (Table 4).

**Table 4:** The details of QTLs associated with yield-related traits under stripe rust stress

QTL name	SNP Marker	Chr.^a^	Position	Alleles^b^	Traits	*P* values (−log)e	Marker R^2^ (%)	Environments	Reference
*QSL.sicau-1AL*	*AX-110408975*	1A	590,994,911	T/C	SL	3.9–4.3	8.3–10.5	CZ17, CZ18, MY17	^d^
*QTKW.sicau-1BL.1* & *QKWPS.sicau-1BL*	*AX-109335890*	1B	670,593,327	A/C	KWPS & TKW	4.0–4.3 & 3.7–4.5	6.7–7.7 & 6.5–8.6	CZ17, MY17	Börner et al. 2002
	*AX-109849833*	1B	670,678,079	G/T	KWPS & TKW	4.2–6.3 & 3.6–4.7	8.9–13.6 & 7.8–10.6	CZ17, MY17	
	*AX-111525685*	1B	670,781,552	C/G	KWPS & TKW	3.3–4.5 & 3.7–3.9	6.8–9.3 & 7.6–8.5	CZ17, MY17	
	*AX-109299717*	1B	670,794,681	G/A	TKW	3.6–4.5	7.6–9.7	CZ17, MY17	
*QTKW.sicau-1BL.2*	*AX-111471952*	1B	681,682,184	A/G	TKW	3.3–3.5	7.1–7.5	CZ17, MY17	Nezhad et al. 2012
*QTKW.sicau-2AS.1*	*AX-108781797*	2A	2,795,252	G/C	TKW	3.2–4.5	6.5–9.5	CZ17, MY17	Cui et al. 2014; Zhang et al. 2014
	*AX-111079592*	2A	3,541,651	G/A	TKW	3.1–3.4	6.4–7.0	CZ17, MY17	
*QTKW.sicau-2AS.2* & *QKWPS.sicau-2AS*	*AX-108919444*	2A	24,057,418	T/G	KWPS & TKW	3.3–5.1 & 3.2–3.4	5.5–9.2 & 5.4–6.2	CZ17, MY17	Zhang et al. 2014
*QSL.sicau-2AL*	*AX-110079477*	2A	432,588,841	T/A	SL	3.2	6.5–6.6	CZ17, MY17	Deng et al. 2017
*QSL.sicau-2DS*	*AX-110647062*	2D	23,025,488	A/T	SL	3.5–3.7	7.1–7.6	CZ17, MY17	Chai et al. 2018
*QSL.sicau-4AS*	*AX-109296730*	4A	68,155,791	C/A	SL	3.5–3.7	7.2–7.5	CZ17, MY17	Luo et al. 2016
*QTKW.sicau-4AL*	*AX-109993853*	4A	538,150,807	G/T	TWK	3.2–3.7	7.7–9.0	CZ17, MY17	^d^
*QKWPS.sicau-4AL.1*	*AX-109830112*	4A	569,760,052	G/A	KWPS	3.4–8.7	7.4–20.4	MY17, CZ18	^d^
*QKWPS.sicau-4AL.2*	*AX-111088719*	4A	620,950,639	T/C	KWPS	3.1–8.5	6.5–20.0	MY17, CZ18	Cui et al. 2013
*QSL.sicau-5AL.1*	*AX-109624254*	5A	595,708,738	G/A	SL	3.3–4.5	7.8–9.2	CZ17, CZ18	Gao et al. 2015
	*AX-110717909*	5A	595,950,156	C/A	SL	3.1–3.4	7.7–8.0	CZ17, CZ18	
*QSL.sicau-5AL.2*	*AX-110521338*	5A	621,939,257	T/C	SL	3–3.4	7.4–7.6	MY17, CZ18	Liu et al. 2014

^a^,Chromosome; ^b^,The alleles marked with underline are favorable alleles

^d^, the potentially novel QTL

*SL* Spike length, *KWPS* Kernel weight per spike, *TKW* Thousand-kernel weight

*CZ17* Chongzhou 2017, *MY17* Mianyang 2017, *CZ18* Chongzhou 2018

### Putative candidate genes of the three potentially novel QTLs

Based on the Chinese Spring reference RefSeq v1.0 (IWGSC) and RefSeq Annotation v1.1 [26], 59, 29 and 33 genes included in *QSL.sicau-1AL*, *QTKW.sicau-4AL* and *QKWPS.sicau-4AL.1* region were selected. Of them, 21 candidate genes were predicted to be involved in the regulation of phytohormone, cell division and proliferation, meristem development, plant or organ development, and carbohydrate transport (Additional file 6).

## Discussion

### Characterization of yield-related traits under stripe rust stress

We evaluated 244 wheat accessions in three field environments under stripe rust stress (CZ17, MY17 and CZ18) and two sites without inoculating *Pst* (CZ17ck and CZ18ck) in Sichuan. The seven yield-related traits displayed significant differences between 2017 and 2018 except KWPS. FTN, SL, KPSl and KPS in 2017 performed better than in 2018, while SlPS, TKW and SlC in 2018 were better than in 2017. Different traits are formed in different growth periods and required and also affected by different growth conditions, such as water, temperature, soil fertility, light and others. Compared the weather conditions during the wheat growing seasons between 2017 and 2018, we considered temperature was the main factor for the difference in FTN between the 2 years (Additional file 7). From the three-leaf stage to the beginning of stem-elongation in the vegetative growth period was the important time to produce tillers [28], and one of the key factors is temperature. Temperatures below 3 °C are not good for tiller development, and the optimal range is 13–18 °C [29, 30]. Wheat in Sichuan Province is sowed from late October to early November, and the vegetative growth is about from planting time to the following March. The weather conditions from December, 2016 to February, 2017 (CZ17 and MY17) were warm, but the temperatures in December, 2017 to February, 2018 (CZ18) were lower than in CZ17 and MY17. The lowest temperature in CZ18 was less than 0 °C (Additional file 7), which seriously reduced the tiller number. Thus, we speculated that the warm weather was the main factor for the higher number of fertile tillers during the vegetative growth period from 2016 to 2017.

Yield-related traits SlPS, TKW and SlC exhibited relatively higher broad-sense heritabilities, while FTN and KPSl showed lower broad-sense heritabilities (Table 1). The results indicated that the environments had a great influence on FTN and KPSl, but less effect on SlPS, TKW and SlC. In other words, SlPS, TKW and SlC were more stable than FTN and KPSl. Consistent with many other reports, FTN had a low heritability and was strongly influenced by environments [5, 9, 31] and KPSl also displayed relatively low heritability, whereas SlPS, TKW and SlC had relatively higher heritabilities [3, 9, 32–34].

The Shannon-Weaver diversity indices reflected the phenotypic diversity to some extent. KPS and TKW showed the highest phenotype diversities and FTN and SlPS displayed the lowest diversities in this study, which were consistent with the reports of Li et al. [35] and Liu et al. [36]. A high phenotypic diversity is beneficial to phenotypic improvement in breeding. As the important components of yield, KPS and TKW exhibited high phenotypic diversities, and accessions with favourable alleles for these traits can be used as elite germplasm for breeding whet cultivars high yield potential.

Compared with the non-inoculation control experiments (CZ17ck and CZ18ck), the accessions under stripe rust stress (CZ17, MY17 and CZ18) exhibited lower KPS, KWPS and TKW. The further comparison between the non-inoculation control and *Pst*-inoculation indicated that many accessions exhibited significantly lower KPS, KWPS and TKW, but higher FTN under stripe rust stress. However, stripe rust did not significantly affect SL, SlPS, KPSl and SlC. The resistant accessions exhibited higher mean values of yield-related traits than susceptible accessions no matter inoculated or not inoculated indicating that stripe rust resistance protects most of the yield-related traits.

Interestingly, both resistant and susceptible accessions under stripe rust stress exhibited higher FTN. As we discussed above, FTN is mainly determined during the vegetative growth period from the three-leaf stage to the beginning of stem-elongation stage [28]. This period is prior to the *Pst* inocultion. So, we speculated that the stripe rust should not have significant effects on FTN. There were many reports also demonstrated that the stripe rust didn’t affect tiller number [37, 38]. The differences in FTN between the control and *Pst*-inoculation fields could be due to other conditions such as weather, water, and soil fertility rather than stripe rust.

There is no doubt that stripe rust can reduce yield, especially the KPS, KWPS and TKW [39–41]. In the present study, the values of KPS, KWPS and TKW of resistant accessions under stripe rust stress were reduced by 1.5, 8.5 and 5.6%, while those of susceptible accessions under stripe rust stress were reduced 2.7, 11.8 and 10.2% separately. Thus, susceptible accessions had more serious reduction by stripe rust than resistant accessions. In other words, resistance can effectively reduce the losses of KPS, KWPS and TKW under stripe rust stress. We inoculated wheat plants with *Pst* around the shooting stage in January and rust appeared on flag leaves at the heading stage, and reached the highest severity around the anthesis to grain filling stage. The anthesis stage is the important time to product kernels (e.g. KPS) [42] and the grain filling period is the key time to determine the kernel weight (e.g. KWPS and TKW) [43]. The *Pst* pathogen produced abundant urediniospore during the flag-leaf stage, and thus reduced the photosynthetic area, which caused the decrease of sugar production [44]. The decrease of sugar supply to the spike results in the fewer grains and smaller grains, and thus exhibited the lower KPS, KWPS and TKW.

Spikes are mainly produced from the 4-leaf stage to the heading stage, and thus SlPS is mainly determined around the 5-leaf stage to 6/7-leaf stage [28]. SlC was calculated by dividing SL by SlPS. In our study, both SL and SlPS might just escape from the major damage period of stripe rust, and thus, did not show significant differences.

The yield is the complex and comprehensive trait, which was affected by many factors. The degree of *Pst* infection, the time of the *Pst* infection, the weather, the water, the soil fertility, even the personal error for measurement, many parameters like above may affect our study. But most of all, we can be sure the infection of stripe rust can result in the decrease of KPS, KWPS and TWK in this study, which were the important components to results in the yield loss.

### Landraces as elite germplasm for breeding

The 244 accessions used in this study, including 79 landraces and 165 cultivars, belong to two different germplasm resources in Sichuan Province. The classification based on the Q-matrix with Bayesian model-based clustering also clearly divided the 244 accessions into two sub-populations. Except one cultivar, accessions in sub-population 1 were all landraces, whereas those in sub-population 2 were primarily cultivars. Obvious distinctions were found in both phenotypes and genotypes between landraces and cultivars. Therefore, the utilization of these landraces should broaden the genetic background in the wheat breeding programs in Sichuan Province.

The comparison analysis for the eight yield-related traits under stripe rust stress between landraces and cultivars showed the significant differences in FTN, SlPS, KPSl, KWPS, TKW and SlC (Table 2). The landraces showed higher FTN, SlPS and SlC than cultivars. Nevertheless, the cultivars had higher KPSl, KWPS and TKW than the landraces. Besides, the landraces exhibited higher diversities in FTN, SL, SlPS and SlC based on the Shannon-Weaver diversity index, while the cultivars displayed higher diversity in KPSl, KWPS and TKW. The wheat landraces may have been shaped by traditional growth practices, while the cultivars have been developed for adapting the local cropping systems. The higher adaptability to different environments, diversity and inheritability are the basic characteristics of landraces [45]. The cultivars were bred by human-mediated selection mainly aiming at achieving high-yield. As one of the three yield components, kernel weight (KWPS and TKW) has been the main target of breeding. The higher KWPS and TKW of the tested cultivars were the outcomes of yield breeding for cultivars. Different from the pursuit of high yield in cultivars, the wheat landraces mainly selected by local farmers, they reserved seeds for planting, the plants with more seeds were their targets, and thus exhibiting higher FTN, SlPS and SlC and higher diversities for these traits. The traits with higher diversities are easy to modify in breeding. Many studies have demonstrated that landraces are excellent germplasm sources, especially for abiotic and biotic stresses [46–50]. Landraces also have many elite yield-related genes [51–54]. Many Chinese wheat cultivars have been developed using landraces, such as Bima 1, Shannong 205, Wuyimai, and Yulin 3 [55, 56]. The represent study provides additional evidence for taking the advantages of landraces with favourable alleles for yield-related traits under stripe rust stress.

### Markers associated to yield-related traits

Here, we identified 13 QTLs associated with SL, KWPS and TKW, which were located on 1AL, 1BL, 2AS, 2AL, 2DS, 4AS, 4AL and 5AL. The QTLs associated with SL, KWPS and TKW was named as *QSL.sicau*, *QKWPS.sicau* and *QTKW.sicau*, respectively (Table 4). Compared the physical locations of QTLs in this study with reported QTLs or genes based on the Chinese Spring reference RefSeq v1.0 [26], three potential novel QTLs were identified. They were *QSL.sicau-1AL*, *QTKW.sicau-4AL* and *QKWPS.sicau-4AL.1*, which were located at different physical positions from previously reported genes related to SL, TKW and KWPS.

The six QTLs were identified associated with SL, including one potentially new (*QSL.sicau-1AL*) and five previously reported QTLs. *QSL.sicau-2AL* was located around the position of 432.58 Mb at 2A, which was the same as *QSl.sdau-2A* [57]. *QSL.sicau-2DS* overlapped with *QPht/SL.cau-2D.2* [58], and the *QSL.sicau-4AS* was covered by *QSl.sau-4A* [59]. Two QTLs were located at 5AL. One was *QSL.sicau-5AL.1*, which was the same as *QSL.caas-5AL* that was flanked by marker *JD_c15758_288* and *BS00041911_51* [32], and another was *QSL.sicau-5AL.2*, which overlapped with *QSl-5A1* that was flanked by SSR marker *Xbarc261* and *Xbarc151* [60]*.*

Three QTLs were associated with TKW and two with KWPS. *QTKW.sicau-4AL* and *QKWPS.sicau-4AL.1* were potentially new based on their physical locations. *QTKW.sicau-1BL.2* was located in the distal region of 1BL was covered by *QTgw.ipk-1B-FS4* [61]. *QTKW.sicau-2AS.1* was mapped on the short end of 2AS, which was overlapped with *QTkw-2A.2* [9] and *Qtkw2A-2* [62]. *QKWPS.sicau-4AL.2* associated with KWPS was a major QTL, with up to 20% PVE. This QTL was covered by *QKwps|Tkw-WJ-4A.1* [63].

We also consistently detected two QTLs associated with both TKW and KWPS. *QTKW.sicau-1BL.1* and *QKWPS.sicau-1BL* were located in the same region around 670 Mb. They were overlapped with *Qgwe.ipk-1B* that was associated with KWPS [64]. However, there are no reports on the association of this QTL with TKW. Our results indicated that this QTL is also related to TKW. In addition, *QTKW.sicau-2AS.2* and *QKWPS.sicau-2AS* were also mapped at the same position of 24.05 Mb, which was very close to *Qtkw2A-1* [62]. *Qtkw2A-1* associated with TKW but not KWPS [62]. We found that this QTL is related to both KWPS and TKW.

We identified *Qyrsicau-1BL.1* around the position of 670 Mb that was associated with stripe rust IT and DS [27], which belonged to the same QTL block of both *QTKW.sicau-1BL.1* and *QKWPS.sicau-1BL*. These results indicate that this QTL block around the position of 670 Mb on 1BL confers stripe rust resistance, and thus related to KWPS and TKW under stripe rust stress in the present study. In addition, *Qyrsicau-1BL.2* around the region of 681 Mb associated with stripe rust IT [27] was the same as *QTKW.sicau-1BL.2* which was also associated with TKW in this study. This is another QTL block conferring stripe rust resistance and thus associated to TKW. These two QTLs might be with pleiotropy were both located on 1BL and just 11 Mb apart. Thus, 1BL harbours numerous QTLs for stripe rust resistance and other traits.

### Candidate genes for the three potentially novel QTLs

A total of 121 genes were selected for the analyses of candidate genes of the three potential novel QTLs. Of these genes, 11, 3 and 7 candidate genes were identified for *QSL.sicau-1AL*, *QTKW.sicau-4AL* and *QKWPS.sicau-4AL.1*, respectively (Additional file 6). Eleven presumptive candidate genes (*TraesCS1A02G439500*, *TraesCS1A02G440000*, *TraesCS1A02G442400*, *TraesCS1A02G443700*, *TraesCS1A02G444100*, *TraesCS1A02G444500*, *TraesCS1A02G444700*, *TraesCS1A02G445100*, *TraesCS1A02G445200*, *TraesCS1A02G445300* and *TraesCS1A02G445400*) were speculated to exist in *QSLsicau-1AL*. *TraesCS1A02G439500* is homologous to *Arabidopsis* gene *EAF1B* (early flowering 1B) which involved in the regulation of transition from vegetative to reproductive phase [65] and the regulation of photoperiodism [66]. The period from vegetative to reproductive growth is important time for spike development in wheat, and the spike development is sensitive to light [28, 67–69]. *TraesCS1A02G440000* is aligned with rice gene *GH3.8* (Probable indole-3-acetic acid-amido synthetase), that is the auxin-responsive gene [70]. Auxin is an important hormone in plant development and we considered the homologous gene in wheat of auxin-responsive gene *GH3.8* associates with spike development and affects the SL. *TraesCS1A02G442400* is an uncharacterized protein in wheat and orthologous with *Arabidopsis* gene *BTAF1* (TATA-binding protein-associated factor 1) involved in the positive regulation of shoot apical meristem development [71]. The shoot apical meristem is responsible for the initiation of many organs, such as nodes, leaves, spike, and inflorescence [72]. Here, we speculate that shoot apical meristems also play an important role in spike development. *TraesCS1A02G443700* is the U6 snRNA-associated Sm-like protein *LSM8*, its orthologous gene is *LSM8* in *Arabidopsis*, which plays a critical role in the regulation of development-related gene expression [73]. The *LSM8* in wheat may also regulate the expression of spike development-related gene. *TraesCS1A02G444500* is homologous to gene *BAM2* (derived from barely any meristem 1) in *Arabidopsis*, which involved in the cell division and differentiation, floral organ development, gametophyte development and regulation of meristem growth [74, 75]. The cell division and differentiation and meristem growth are all associated with the plant development. Hord et al. [76] reported the *BAM1*/*BAM2* receptor-like kinases regulate the early anther development through cell division and differentiation. The spike development along with the anther development, maybe also regulated by the *BAM2* in wheat. *TraesCS1A02G444700* is orthologous with the aspartic proteinase *NANA* in *Arabidopsis*. It’s involved in the carbohydrate metabolic process, maintenance of shoot apical meristem identity and general morphology and development [77, 78]. The carbohydrate metabolic can provide the energy for spike development. The shoot apical meristem and general morphology and development all maybe involved in the spike development [79]. *TraesCS1A02G444100*, *TraesCS1A02G445100*, *TraesCS1A02G445200*, *TraesCS1A02G445300*, and *TraesCS1A02G445400* were all aligned with rice gene *RR42* (Two-component response regulator 42), which is involved in the cytokinin-activated signaling pathway and phosphorelay signal transduction system [80, 81]. Cytokinin is the classic plant growth phytohormones and functions to promote the cell division and cell differentiation, which may contribute to the spike development in wheat.

There were three putative candidate genes for *QTKW.sicau-4AL*, *TraesCS4A02G229100*, *TraesCS4A02G229600*, and *TraesCS4A02G229700*. *TraesCS4A02G229100* is the auxin regulated gene involved in organ size (*TaARGOS-A*). Zhao et al. [82] studied the *TaARGOS* influenced plant growth and stress tolerance, and the GO annotation showed it involved in the positive regulation of organ growth. Its homologous gene *ARGOS* in rice responds to auxin stimuli, positively regulate cell and organ growth [83]. Auxin is an important hormone in plant development. In *Arabidopsis*, *ARF2* functions as an auxin response factor playing a vital role in determining final size of the seed [84]. In rice, auxin transporters can affect kernel size and increase the TKW [85]. We speculate that *TraesCS4A02G229100* as an *ARGOS* gene also respond to auxin and regulated the organ (spike or grain) growth in wheat. *TraesCS4A02G229600* and *TraesCS4A02G229700* were all orthologous with *Arabidopsis* gene *At2g43860*, as a polygalacturonase, involved in the carbohydrate metabolic process [86]. Carbohydrate is a main product of photosynthesis, and it can be transported to spikes for kernel growth and further determining kernel size and weight [10, 87].

Seven putative candidate genes, *TraesCS4A02G255800*, *TraesCS4A02G256500*, *TraesCS4A02G256700*, *TraesCS4A02G257100*, *TraesCS4A02G257200*, *TraesCS4A02G257700*, and *TraesCS4A02G258000*, were detected in *QKWPS.sicau-4AL.1*. *TraesCS4A02G255800* is homologous to the transcription factor *bHLH74* (basic helix-loop-helix 74) in *Arabidopsis*. It involved in cell elongation, plant development and triggering flowering in response to blue light [88–90]. In rice, the homologous gene *bHLH74* can regulate the cell elongation and finally control the grain size [91]. Grain size, as an important yield component, may also be regulated by *bHLH74* homologous gene in wheat. *TraesCS4A02G256500* is aligned with rice gene *ACC1* (1-aminocyclopropane-1-carboxylate synthase 1). The *ACC1* is a kind of synthase, which could catalyze the formation of 1-aminocyclopropane-1-carboxylate that’s a direct precursor of ethylene in higher plants. Ethylene is well known as the effect on fruit ripening and organ abscission. Yang et al. [92] found the abscisic acid and ethylene in wheat grains can respond to the drought during the grain filling. Naik and Mohapatra (2000) [93] reported the ethylene had effect on the grain filling of basal rice kernels. The grain filling is the important stage to determine the kernel yield in wheat. We speculated the homologous gene *ACC1* in wheat can regulate the ethylene as well and further impact the kernel yield. *TraesCS4A02G256700* is the gene *Wknox1a*, which mainly expresses in shoot apical meristem-containing shoots and young spikes in wheat [94]. *Wknox1a* is aligned with rice gene *OSH1*, which affects the inflorescence morphology [95]. In addition, *OSH1* regulates the auxin mediated signalling pathway [96], and as a member of the *KNOX* protein family, it plays an important role in shoot apical meristem maintenance [97]. Auxin and shoot apical meristems are all involved in the inflorescence development and further affect kernel traits [72, 84]. *TraesCS4A02G257100* was the homolog of GDP-mannose transporter *GONST1* in *Arabidopsis*. One of the important functions of *GONST1* is carbohydrate transport [98]. It is involved in transporting carbohydrates from leaves to spikes, a vital activity to support kernel growth. The condition of the kernel growth would affect KWPS in wheat. *TraesCS4A02G257200* is orthologous with *Arabidopsis* gene *AMSH3. AMSH3* is essential for plant growth and development [99]. KWPS is determined by many aspects of growth and development, such as spike development, spikelet and kernel development. *TraesCS4A02G257700* is the inositol-tetrakisphosphate 1-kinase and the GO annotation showed it’s involved in inositol trisphosphate metabolic process. Its orthologous gene *ITPK1* in maize, also involved in inositol trisphosphate metabolic process, participates in phytic acid biosynthesis in developing seeds. Phytic acid is an important storage form of phosphorus in cereal grains [100], which may influence the kernel yield directly. *TraesCS4A02G258000* is homologous to COMPASS-like *H3K4* histone methylase component *WDR5a* (WD40-REPEAT 5a) in *Arabidopsis*. It involved in vegetative to reproductive phase transition of meristem, and expressed in developing embryos and endosperms, shoot and root apical regions [101, 102]. The differentiation of meristem from vegetative to reproductive growth is important time for the initial of spikelet development in wheat and further impacts the yield components [28, 67].

Although the putative candidate genes were analysed based on collinearity analysis with the limited known information about the gene/protein function, it still provides us much important information to identify the possible candidate genes. We will further study these candidate genes by genetic mapping or reverse genetics in the future.

## Conclusions

Molecular marker-assisted breeding is an effective and environment-friendly way to improve yield and disease resistance. In this study, we collected 244 accessions with high diversity from Sichuan, the phenotypic comparison analysis between resistance and susceptible accessions with or without *Pst* inoculation showed that the resistance accessions had much reduced yield losses (KPS, KWPS and TKW). Combined with 44,059 effective markers, we identified three potential novel loci and 16 favourable alleles through GWAS analysis, providing reliable markers and elite genetic stocks for molecular marker-assisted breeding.

## Materials and methods

### Plant materials

A total of 244 wheat accessions (Additional file 2) were used in this study, including 79 landraces and 165 cultivars which have been used or developed by different breeding programs in Sichuan Province since 1997.

### Evaluation of yield-related traits and stripe rust infection type

The 244 wheat accessions were evaluated in two locations in Sichuan with different years but all under stripe rust stress: Chongzhou (30°33′37.3″ N, 103°38′45.4″ E, elevation 513 m) in 2017 (CZ17) and 2018 (CZ18); Mianyang (31°23′N, 104°49′E, elevation 440 m) in 2017 (MY17). All experimental fields were inoculated with mixed urediniospores of the local *Pst* races, including CYR32, CYR33, CYR34, G22–14, Su11–4, Su11–5, Su11–7 [27]. The 244 wheat accessions were also planted in different fields in Chongzhou (30°33′46.3″N, 103°38′38.5″E, elevation 514 m) without inoculation in 2017 (CZ17ck) and 2018 (CZ18ck). This field is about 0.5 km away from the inoculated field and sprayed with fungicide 25% Triadimefon at the rate of 0.2 kg/ha at the early infection stage (around booting stage for twice) and heading stage. In all test environments, twenty seeds of each accession were evenly planted in a 2 m row with a 0.3 m between rows. The accessions with three replications planted in each location were evaluated for eight yield-related traits: FTN, SL, SlPS, KPSl, KPS, KWPS, TKW, and SlC which was calculated by dividing SL by SlPS. All of the traits were measured on five randomly selected plants for each accession at harvest. The rule of identification of infection type (IT) for stripe rust was the same as Ye et al. [27]. In order to reduce the environmental impacts on yield-related traits, the best linear unbiased prediction (BLUP) values were calculated based on linear model using the lme4 package in the R program [103]. The broad-sense heritability (*H*^*2*^) estimates for each of the yield-related traits were calculated across all test environments using formula *H*^*2*^ = V_G_/(V_G_ + V_E_) using the lme4 package [103], where V_G_ and V_E_ are the genotypic and environmental variances, respectively [104]. The phenotypic variation was determined by the range, mean, standard deviation (STDEV) and coefficient of variation (CV) for each trait and BLUP value. The Pearson correlation coefficient and the *t*-test were achieved using SPSS 20.0 (IBM Corp., Armonk, NY, USA). The Shannon-Weaver diversity index (*H′*) was calculated for each trait using the BLU*P* values [105].

### Genotyping analysis

The genomic DNA was extracted from the mixed leaves collected from 5 one-week-old seedings using the plant DNA kits (Biofit Co., China) for each accession. A total of 244 DNA samples were genotyped using the 55 K SNP array (Affymetrix Axiom Wheat55K) at the China Golden Marker Biotechnology Company Ltd. (Beijing, China). The effective markers used for further analyses were selected with missing values ≤10% and minor allele frequency (MAF) ≥ 5%.

### Population structure, kinship and linkage disequilibrium analysis

The population structure (Q-matrix) was analysed using software STRUCTURE v2.3.4 with Bayesian model-based clustering [106]. Five independent STRUCTURE runs were performed with the K from 2 to 10 using the admixture model with 100,000 replicates for burn-in length and 100,000 replicates for Markov chain Monte Carlo (MCMC) iterations. The optimal K value was chosen using the ΔK method in web-based software STRUCTURE HARVESTER [107]. The kinship (K-matrix) was estimated between pairs of accessions as a measure of relatedness based on the identity-by-state (IBS) method using TASSEL v5.2.38 [108]. The pairwise measure of linkage disequilibrium (LD) was estimated as squared allele frequency correlation (*r*^*2*^) between pairs of intra-chromosomal markers with known chromosomal position using TASSEL v5.2.38 [108]. Significant pair-wise markers were chosen using the threshold pDiseq < 0.001 and *r*^*2*^ > 0.1 [108]. The LD decay plot and half decay distance were generated with the *r*^*2*^ values and the distances between markers using the ggplot2 package in the R program [109]. All high confidence associated loci in the half decay distance region on the same chromosome were defined as the same QTL block.

### Genome-wide association analyses

Combining the yield-related traits under stripe rust stress with 44,059 effective SNP markers, GWAS analyses were performed on the 244 accessions using software TASSEL v5.2.38 based on the mixed linear model (MLM) with Q and K as covariates [108, 110, 111]. For GWAS results, a threshold *P*-value of 0.001 (−log_10_ *P* = 3) was considered as the significant association markers. To make significant associated loci more reliable, the high confidence associated loci were selected for further analyses. The high confidence associated loci should be the significant association loci which must be detected in at least two test environments. The associated loci with related traits were visualized with Manhattan plots with *P* values using the ggplot2 package in the R program [109].

### Analyses of high confidence significant associated loci

There are many QTLs associated with yield-related traits previously reported. In order to identify potentially novel loci, the physical location of each QTL was determined based on the high-quality Chinese Spring reference IWGSC RefSeq v1.0 [26] using software BLAST+ v2.7.1 [112].

### Analyses of putative candidate genes in three potentially novel QTLs

By referencing the Chinese Spring reference genome (IWGSC RefSeq v1.0) and RefSeq Annotation v1.1 [26], the genes included in three potentially novel QTLs were selected based on the LD decay distance 2.12 Mb. The collinear analysis was carried out using online BLAST at the EnsemblPlants website (https://plants.ensembl.org/Multi/Tools/Blast) with default parameters.

## Additional files

Additional file 1: Pearson coefficient analysis for eight yield-related traits among multiple environments (XLSX 11 kb)

Additional file 2: Two hundred and forty-four wheat accessions used in this study and the evaluation of their yield-related traits and infection type (IT) in multiple environments (XLSX 106 kb)

Additional file 3: The 44,059 effective 55 K SNP markers used in this study (ZIP 2718 kb)

Additional file 4: Phenotypic variations for two sub-populations based on Q-matrix (XLSX 10 kb)

Additional file 5: The distribution of the favourable alleles in 244 wheat accessions (XLSX 25 kb)

Additional file 6: The candidate genes of three potential novel QTL (XLSX 12 kb)

Additional file 7: Temperatures during the wheat growth seasons in three environments. The plant growth season was from October to the following May. The lowest temperature in CZ18 was lowest among all three environments and lower than 0 °C from December to following February. CZ17 = Chongzhou 2017; MY17 = Mianyang 2017; CZ18 = Chongzhou 2018 (PDF 74 kb)

## Data Availability

Not applicable.

## Abbreviations

BLUP: Best linear unbiased prediction
CV: Coefficient of variation
CZ: Chongzhou
FTN: Fertile tiller number
GWAS: Genome-wide association study
*H′*: Shannon-Weaver diversity index
*H*^*2*^: Broad-sense heritability
IT: Infection type
KPS: Kernel number per spike
KPSl: Kernel number per spikelet
KWPS: Kernel weight per spike
LD: Linkage disequilibrium
MY: Mianyang
*Pst*: *Puccinia striiformis* f. sp. *Tritici*
QTL: Quantitative trait locus
SL: Spike length
SlC: Spikelet compactness
SlPS: Spikelet number per spike
STDEV: Standard deviation
TKW: Thousand-kernel weight
