# Supplementary figures and images for: Genome-wide association study reveals new loci for yield-related traits in Sichuan wheat germplasm under stripe rust stress
Source: BMC Genomics. 2019 Aug 8;20:640. doi: 10.1186/s12864-019-6005-6 (PMC6688255; doi:10.1186/s12864-019-6005-6)

**Average temperature**

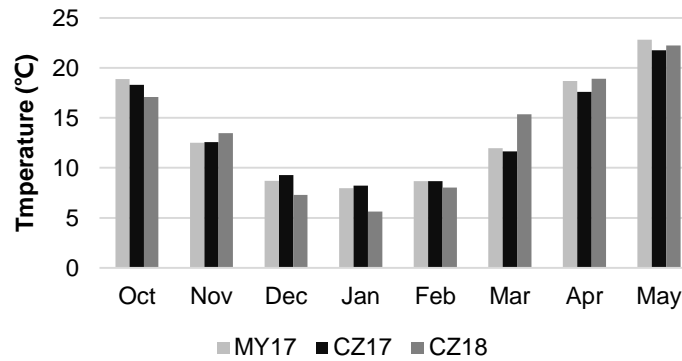

**Highest temperature**

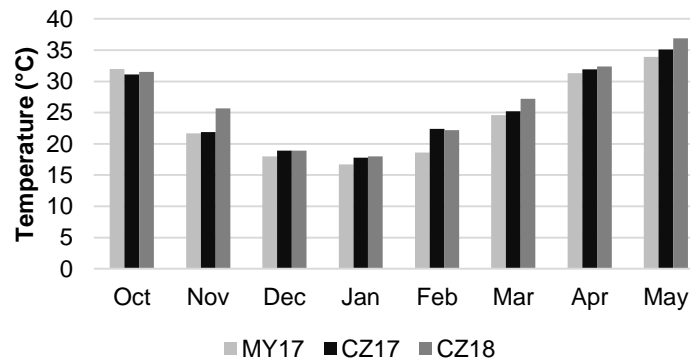

**Lowest temperature**

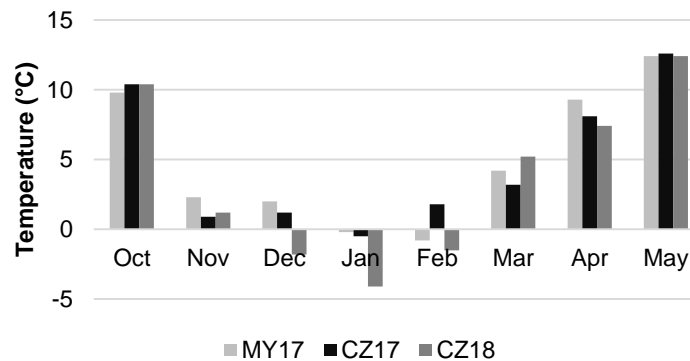

Supplement: Supplementary file 7 — Temperatures during the wheat growth seasons in three environments. The plant growth season was from October to the following May. The lowest temperature in CZ18 was lowest among all three environments and lower than 0 °C from December to following February. CZ17 = Chongzhou 2017; MY17 = Mianyang 2017; CZ18 = Chongzhou 2018 (PDF 74 kb) [file 12864_2019_6005_MOESM7_ESM.pdf]
